# Intervention Mechanism of Niao Du Kang Mixture on the EMT Process of Peritoneal Fibrosis Based on the Wnt/*β*-Catenin Signaling Pathway

**DOI:** 10.1155/2022/2089483

**Published:** 2022-11-07

**Authors:** Lin Huang, Zhengxinyi Wang, Yanlin Li

**Affiliations:** ^1^Zhongshan Hospital of TCM, Zhongshan 528400, Guangdong, China; ^2^Zhongshan Hospital of TCM, Guangzhou University of Traditional Chinese Medicine, Zhongshan 528400, Guangdong, China

## Abstract

**Methods:**

Quantification of 24-hour urine protein (24 h-Upro) and serum creatinine (Scr) levels was performed. The protein and mRNA expression levels of E-cadherin, *α*-SMA, collagen I, *β*-catenin, Wnt-1, and LEF-1 in peritoneal tissue were measured. In addition, the pathological morphology and ultrastructure of peritoneum were observed.

**Results:**

After 5/6 nephrectomy + high glucose peritoneal dialysate + lipopolysaccharide (LPS) treatment, the Scr and 24 h-Upro of rats increased compared with normal rats, and the peritoneal tissue was damaged and thickened, showing fibrotic changes. Compared with the model group, the Scr and 24 h-Upro levels and the levels of E-cadherin, *α*-SMA, Wnt-1, collagen I, and LEF-1 protein expression in each Niao Du Kang mixture dosage group decreased. The protein expression of *β*-catenin and the mRNA expression of E-cadherin, *α*-SMA, Wnt-1, collagen I, *β*-catenin, and LEF-1 decreased in the high and medium Niao Du Kang mixture dose groups. Hematoxylin and eosin staining showed that the peritoneum of the rats was not only thicker in the model group than in the sham operation group (*P* < 0.01) but also accompanied by apparent inflammatory cell infiltration, tissue edema, and fibrosis. Compared with the model group, all the Niao Du Kang mixture groups demonstrated various degrees of mitigation in peritoneal thickness and fibrosis (*P* < 0.01). The strongest effect was observed in the medium-dose group. Transmission electron microscopy showed that the degree of injury of the peritoneal mesothelial cells was ranked as follows: model group > positive drug group > Niao Du Kang mixture high-dose group.

**Conclusions:**

The Niao Du Kang mixture may effectively decrease the peritoneal thickness and fibrosis degree through its effect on the Wnt/*β*-catenin signaling pathway involved in EMT. The present study provides data that assist in elucidating the potential function of the Niao Du Kang mixture in treating or preventing PF.

## 1. Introduction

Peritoneal dialysis (PD) is one of the replacement therapies for end-stage renal disease. However, peritoneal tissue is exposed to dialysis fluid with poor biocompatibility for a long time, and repeated peritonitis can lead to peritoneal damage, resulting in peritoneal fibrosis (PF) [1], lowering dialysis efficiency, and eventually leading to ultrafiltration failure. Peritoneal fibrosis is closely related to inflammation, angiogenesis, and EMT, and these processes interact. EMT plays an important role not only in organ development and wound healing but also in diseases including cancer and tissue fibrosis in the peritoneum. Mesothelial to mesenchymal transition (MMT) in the peritoneal membrane is indicative of its failure and is a trigger for peritoneal fibrosis. During this transition, mesenchymal cells migrate from the superficial mesenchymal layer to the inferior mesenchymal layer, where they produce the extracellular matrix (ECM) that promotes fibrosis [2]. Activation of the Wnt/*β*-catenin signaling pathway is thought to play a key role in the EMT process [3].

Previous studies have shown that the Niao Du Kang mixture can significantly improve renal function in rats with unilateral ureteral ligation, improve the EMT of renal tubular cells on the ligation side, and significantly reduce the degree of renal interstitial fibrosis in unilateral ureteral obstruction (UUO) rats. The effect is achieved by inhibiting the P38/extracellular regulated protein kinases (ERK) mitogen-activated protein kinases (MAPK) pathway [4]. The Niao Du Kang mixture can inhibit the expression of phosphoinositol-dependent protein kinase 1 (PDPK1) by upregulating the expression of mir-129-5p and then inhibiting the PI3K/AKT pathway to improve renal fibrosis [5]. Additionally, the Niao Du Kang mixture can improve the dialysis efficiency of patients with PD, delay the deterioration of renal function, and inhibit peritoneal fibrosis and renal microinflammation. After three months of clinical observation, it was found that compared with the patients in the peritoneal dialysis group, the levels of fibronectin and transforming growth factor-*β*1 (TGF-*β*1) in peritoneal dialysis fluid and the levels of high mobility group-1 (HMGB1), high-sensitivity C reactive protein (hsCRP), and interleukin-6 (IL-6) in serum were lower in patients in the peritoneal dialysis combined with Niao Du Kang mixture group [6]. However, its mechanism of action remains unclear. In the current study, we used 5/6 nephrectomy + high glucose peritoneal dialysis fluid + lipopolysaccharide (LPS) method to replicate peritoneal fibrosis in a rat model. Niao Du Kang mixture was used as an interventional drug, and we observed its influence on the structure of peritoneal tissue and explored whether its intervention mechanism is related to the Wnt/*β*-catenin signaling pathway.

## 2. Materials and Methods

### 2.1. Experimental Animals

SPF-grade healthy Wistar rats, male, weighing 180–200 g, were purchased from Beijing Weitong Lihua Laboratory Animal Technology Co. Ltd. (laboratory animal certificate number SCXK (Jing) 2016-0006; use of experimental animal permit license number SYXK (Jing) 2016-0007). All operations were carried out in strict accordance with the relevant ethical guidelines for using animals in research and were approved by the ethics committee (approval number: IMPS-EAEP-H-H2018049-01).

### 2.2. Drugs and Reagents

Niao Du Kang mixture (100 ml/bottle, provided by Zhongshan Traditional Chinese Medicine Hospital Affiliated with Guangzhou University of Chinese Medicine, batch number 20200519). Niao Du Qing Granules (Kangchen Pharmaceutical Co., Ltd., batch number 20181168); Penicillin Sodium for Injection (Harbin Pharmaceutical Group Co., Ltd., batch number 18020402-2); Low Calcium Peritoneal Dialysis Solution (lactate-G4.25%) (Guangzhou Baite Medical Products Co., Ltd., batch number G19072412); lipopolysaccharide (Sigma, batch number 039M4004V).

Enhanced BCA protein assay kit, Gallocyanine (Aibixin Biotechnology Co., Ltd., batch number abs9233, SC16); collagen I and Wnt-1 (batch number NC04, MC26); ECL Substrate Kit (Ultra High Sensitivity) (batch number EL2001001); 5x protein loading buffer (Beijing Soleibo Technology Co., Ltd., batch number 20200115); E-cadherin, *α*-SMA, *β*-catenin, LEF-1, GAPDH, and Anti-Rabbit IgG (CST, batch number 202003, 202003, 202002, 202003, 201910, and 201909, respectively); UltraSYBR Mixture (Kang Wei Century, batch number 40222).

### 2.3. Instruments

Monopolar electrocoagulation device (Wuhan Chunguang Medical Beauty Instrument Co., Ltd.); Hitachi 7080 automatic biochemical analyzer (Sekisui Medical Technology Co., Ltd., Japan); Ci-L microscope (Nikon, Japan); ECL gel documentation system (Life Technologies, USA); GET96-PLUS PCR device (Hangzhou Boheng Technology Co., Ltd.); Real-time PCR device (Roche, Switzerland); HT7700 transmission electron microscope (HITACHI, Japan).

### 2.4. Model Preparation and Drug Administration

Eighty male Wistar rats, adaptively fed for seven days, were randomly divided into the sham operation group, model group, positive drug group, and Niao Du Kang mixture high, medium, and low-dose groups. The peritoneal fibrosis model was established by 5/6 nephrectomy + high glucose peritoneal dialysate + lipopolysaccharide (LPS). One week after modeling, 4.25% glucose peritoneal dialysate (30 ml/kg) was intraperitoneally injected once daily for 28 consecutive days, and LPS (0.6 mg/kg) was intraperitoneally injected on days 8, 10, 12, 22, 24, and 26. Intragastric administration of drugs began simultaneously with the intraperitoneal injection of dialysate. The positive drug group received intragastric administration of Niao Du Qing granules, 2.5 g/kg (converted according to the clinical dosage used in humans), which is a traditional Chinese medicine formula that is used for chronic renal failure. The high, medium, and low dosage groups received intragastric administration of the Niao Du Kang mixture at 20, 10, and 5 ml/kg, respectively (relative to 12, 6, and 3 times the clinical dosage used in humans), with a gavage volume of 10 ml/kg. Purified water was provided to the sham operation and model groups that was equal to their volume by gavage for 28 consecutive days.

### 2.5. Indicator Detection

#### 2.5.1. Detection of Serum Creatinine

To induce anesthesia, 45 mg/kg of sodium pentobarbital was injected intraperitoneally. Blood was collected from the abdominal aorta, left standing for 1-2 h, and centrifuged at 3000 revs. min^−1^ for 10 min to separate the serum. The supernatant was collected and used to measure the level of Scr by the enzymatic method.

#### 2.5.2. Detection of 24 h Urine Protein

Metabolic cages were used to measure the amount of protein in rat urine. After dry fasting, urine was collected for 24 h. The total volume of urine within 24 h was recorded, the supernatant was removed after centrifugation, and then, the 24 h-Upro was determined by the midpoint method.

#### 2.5.3. Hematoxylin-Eosin (HE) Staining to Observe the Pathological Morphology of Peritoneal Tissue

The fixed peritoneal tissue was dehydrated, embedded in paraffin, sliced, and stained with HE. The peritoneal thickness was observed and measured under a light microscope. Three peritoneal thicknesses were randomly measured in each visual field and averaged.

#### 2.5.4. Western Blot Detection of Expression of E-Cadherin, *α*-SMA, Collagen I, *β*-Catenin, Wnt-1, and LEF-1 Proteins

An appropriate amount of peritoneal tissue was removed from storage at −80°C, and the tissue was then ground and centrifuged, and the supernatant was aspirated and used to measure the protein concentration. To prepare the samples, 5x protein loading buffer and normal saline were added, and the samples were transferred into sterile centrifuge tubes for protein denaturation. After the stacking gel and separating gel were prepared and constructed, the samples were loaded to carry out sodium dodecyl sulfate-polyacrylamide gel electrophoresis (SDS-PAGE) with constant circulating membrane and skim milk blocking buffer. The ratio of the target band to the internal reference gray value was determined using Image J software to represent the expression level of the tested protein.

#### 2.5.5. Real-Time PCR Detection of E-Cadherin, *α*-SMA, Collagen I, *β*-Catenin, Wnt-1, and LEF-1 mRNA Expression

The total RNA of ileal tissue was extracted with TRIzol reagent, the RNA concentration of each sample was measured, and RNA reverse transcription was performed. The DNA concentration was measured after reverse transcription. Once confirmed, 1 *μ*L of DNA was added to the mixture, and then, the contents were mixed with the centrifuge tube lid closed. The level of mRNA expression in each group was determined using the delta-delta Ct method.

#### 2.5.6. Observation of the Ultrastructure of the Peritoneal Tissue by Transmission Electron Microscope

The peritoneal tissue, fixed in 2.5% glutaraldehyde solution, was removed, embedded, polymerized, sliced, double-stained with uranyl acetate and lead citrate, observed under a transmission electron microscope, and recorded for image analysis.

### 2.6. Statistical Analysis

SPSS 19.0 was used for data analysis. The measurement data are presented as $\bar{x}\pm s$. One-way analysis of variance was used for comparison among groups. *P* < 0.05 indicated that the difference was statistically significant.

## 3. Results

### 3.1. The Effect of the Niao Du Kang Mixture on the Levels of Scr and 24 h-Upro in Peritoneal Fibrosis Rats

Compared with the levels of Scr and 24 h-Upro in the sham operation group, those in the model group significantly increased (*P* < 0.01). Compared with the levels of Scr and 24 h-Upro in the model group, those in the Niao Du Qing granules and Niao Du Kang mixture groups significantly decreased (*P* < 0.01) (see Table 1).

### 3.2. The Effect of Niao Du Kang Mixture on the Pathological Morphology and Thickness of the Peritoneum in Rats with Peritoneal Fibrosis

**Figure 1:**
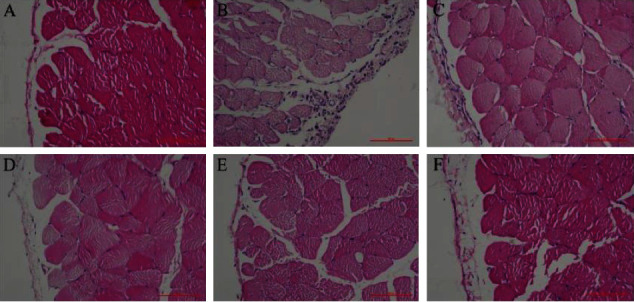
The effect of the Niao Du Kang mixture on the pathological morphology of the peritoneum in rats with peritoneal fibrosis (HE, 200×). (a) Sham operation group. (b) Model group. (c) Niao Du Qing granule group. (d) Niao Du Kang mixture high-dose group. (e) Niao Du Kang mixture medium-dose group. (f) Niao Du Kang mixture low-dose group.

HE staining showed that the peritoneum of the rats in the sham operation group was thinner, and there was no apparent inflammatory cell infiltration or tissue edema. The peritoneum of the rats in the model group was significantly thicker than that of rats in the sham operation group (*P* < 0.01), accompanied by apparent inflammatory cell infiltration, tissue edema, and fibrosis. Compared with the model group, all the groups that received the Niao Du Kang mixture demonstrated various degrees of mitigation in peritoneal thickness and fibrosis (*P* < 0.01). The medium-dose group demonstrated the most optimal effect among all the groups that received the Niao Du Kang mixture (see Figure 1 and Table 2).

### 3.3. The Effect of the Niao Du Kang Mixture on the Expression of E-Cadherin, *α*-SMA, Collagen I, *β*-Catenin, Wnt-1, and LEF-1 Proteins in the Peritoneal Tissue of Peritoneal Fibrosis Rats

**Figure 2:**
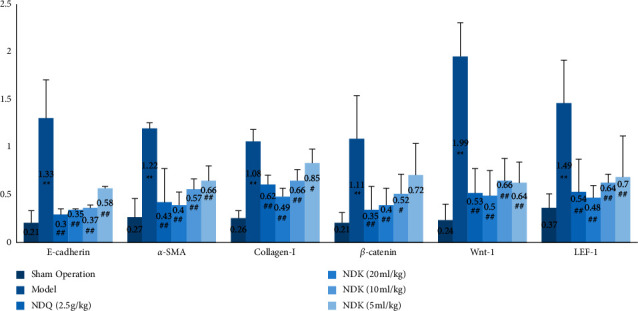
The effect of the Niao Du Kang mixture on the levels of E-cadherin, *α*-SMA, collagen I, *β*-catenin, Wnt-1, and LEF-1 protein expression in the peritoneal tissue of peritoneal fibrosis rats ($\bar{x}\pm s$, *n* = 4). Compared with the sham operation group, ^∗∗^*P* < 0.01; compared with the model group, ^#^*P* < 0.05, ^##^*P* < 0.01.

**Figure 3:**
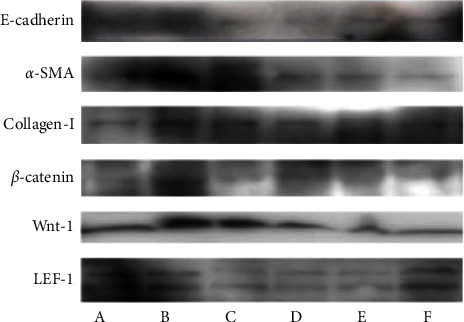
Western blot strip chart. (A) Sham operation group. (B) Model group. (C) Niao Du Qing granule group. (D) Niao Du Kang mixture high-dose group. (E) Niao Du Kang mixture medium-dose group. (F) Niao Du Kang mixture low-dose group.

The levels of E-cadherin, *α*-SMA, collagen I, *β*-catenin, Wnt-1, and LEF-1 protein expression were significantly higher in the model group than in the sham operation group (*P* < 0.01). The levels of E-cadherin, *α*-SMA, collagen I, Wnt-1, and LEF-1 protein expression were significantly lower in the Niao Du Qing granule and Niao Du Kang mixture groups than in the model group (*P* < 0.01, *P* < 0.05). The levels of *β*-catenin protein expression in the Niao Du Qing granule group and the Niao Du Kang mixture high and medium-dose groups were significantly lower (*P* < 0.01, *P* < 0.05) (see Figures 2 and 3).

### 3.4. The Effect of Niao Du Kang Mixture on the mRNA Expression Levels of E-Cadherin, *α*-SMA, Collagen I, *β*-Catenin, Wnt-1, and LEF-1 in Peritoneal Tissue of Peritoneal Fibrosis Rats

**Figure 4:**
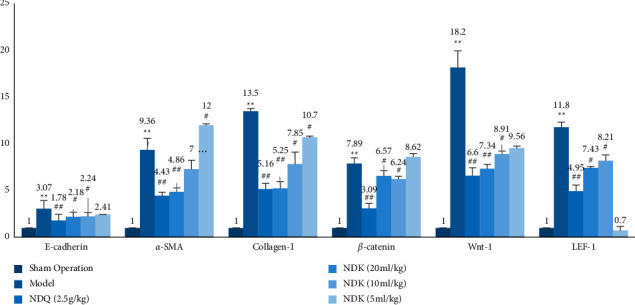
The effect of the Niao Du Kang mixture on the mRNA expression levels of E-cadherin, *α*-SMA, collagen I, *β*-catenin, Wnt-1, and LEF-1 mRNA in the peritoneal tissue of rats with peritoneal fibrosis ($\bar{x}\pm s$, *n* = 4). Compared with the sham operation group, ^∗∗^*P* < 0.01; compared with the model group, ^#^*P* < 0.05, ^##^*P* < 0.01.

The mRNA expression levels of E-cadherin, *α*-SMA, collagen I, *β*-catenin, Wnt-1, and LEF-1 were significantly higher in the model group than in the sham operation group (*P* < 0.01). The mRNA expression levels of E-cadherin, *α*-SMA, collagen I, *β*-catenin, Wnt-1, and LEF-1 were significantly lower in the Niao Du Qing granule and the Niao Du Kang mixture high-dose and medium-dose groups than in the model group (*P* < 0.01, *P* < 0.05) (see Figure 4).

### 3.5. Effects on the Ultrastructure of Peritoneal Tissue

**Figure 5:**
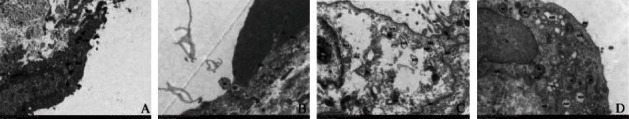
TEM image (7000×). (a) Sham operation group. (b) Model group. (c) Niao Du Qing granule group. (d) Niao Du Kang mixture high-dose group.

Peritoneal mesothelial cells in the model group displayed the most severe damage. The peritoneal mesothelial cells in the positive drug group displayed the second most severe damage. The Niao Du Kang mixture high-dose group displayed the least damage of peritoneal mesothelial cells, in which the cell membrane was intact, the cells were slightly swollen, the basement membrane was intact, and the intercellular space was tight (see Figure 5).

## 4. Discussion

PF occurs through two pathological mechanisms: retroperitoneal fibrosis and infection and inflammation caused by non-biocompatible solutions. The peritoneum is a single layer of continuous epithelial cells with regenerative properties, namely, peritoneal mesothelial cells (PMCs). In the early stage of PF, PMCs possess enhanced abilities for invasion and migration and transform into myofibroblasts, which induce the deposition of the ECM in the mesothelial area. During a long period of PD therapy, the integrity of the patient's peritoneum is compromised. Myofibroblasts have been observed in the PF experimental model and in biopsy specimens of PD patients, and EMT-transformed myofibroblasts have also been observed in the peritoneal mesothelial region [7, 8]. Aroeira et al. used glucose dialysate to establish a PF model, in which partial exfoliation of PMCs was observed after two weeks. PF and angiogenesis occurred after five weeks, accompanied by peritoneal ultrafiltration failure. Fluorescent cytokeratin and *α*-SMA were used to highlight the characteristics of PMCs and myofibroblasts, demonstrating that PMCs undergo EMT [9].

TGF-*β*1 is considered the most crucial cytokine involved in organ fibrosis during EMT. TGF-*β*1 can activate various signaling pathways in the body to exert biological effects, with the TGF-*β*1/Smads pathway being the most important one. In 2002, it was first discovered in a rat renal fibrosis model that Wnt/*β*-catenin signal transduction regulates organ fibrosis [10]. Studies have shown that the Wnt/*β*-catenin and TGF-*β*1/Smads signaling pathways interact and inter-regulate. TGF-*β*1 significantly upregulates Wnt/*β*-catenin and its downstream target genes and silences *β*-catenin. Dkkl inhibits the hypersecretion of TGF-*β*1 and affects the transcription of the Smad protein, meaning that the fibrotic process that is regulated by TGF-*β*1 requires *β*-catenin signaling activation [11]. In an obstructive renal injury rat model, the expression of most Wnt proteins was upregulated, which produced a large volume of FZD receptors and Wnt antagonists. Furthermore, *β*-catenin transcription was enhanced, and nuclear translocation was observed in the renal tubular epithelial cells and renal interstitium. Obstructing the Wnt/*β*-catenin signaling pathway can significantly downregulate the expression of *α*-SMA, which results in the inhibition of EMT and renal fibrosis [12]. The purpose of the current study is to investigate whether the intervention mechanism of the Niao Du Kang mixture that affects the EMT process of peritoneal fibrosis is related to the Wnt/*β*-catenin signaling pathway based on animal experiments.

The Niao Du Kang mixture is composed of Chinese rhubarb (Da Huang, Rhei Radix et Rhizoma/(Rheum undulatum var. longifolium C. Y. Cheng and T. C. Kao), burnet-bloodwort (Di Yu, Sanguisorbae Radix (*Sanguisorba officinalis* L.), safflower (Hong Hua, Carthami Flos/*Carthamus tinctorius* L.), Chinese salvia (Dan Shen, Salviae Miltiorrhizae Radix et Rhizoma/*Salvia miltiorrhiza* Bunge), and astragalus (Huang Qi, Astragali Radix/*Astragalus membranaceus* (Fisch.) Bunge). The functions of the mixture include invigorating Qi for consolidating the exterior of the body, clearing away heat and toxic materials, inducing diuresis to alleviate edema, promoting blood circulation, and removing blood stasis. This formula protects the residual kidney function and delays the progress of kidney diseases.

Experimental studies have shown that [13] astragaloside IV (AS-IV) derived from *A. membranaceus* attenuates high glucose-induced EMT by inhibiting the TGF-*β*1/Smads pathway in renal tubular epithelial cells (PTCs). In mice with UUO, emodin can downregulate the expression of TGF-*β*1 and P-SMad3, decrease the production of fiber markers (including collagen I, collagen III, *β*-catenin, and *α*-SMA), and relieve renal interstitial fibrosis [14]. Salvia may inhibit the expression of collagen and fibronectin that are related to the TGF-*β*1/Smad pathway, thereby decreasing the hypertrophy and dilation of renal tubules and glomeruli [15]. One of salvia's active ingredients, tanshinone, was shown to attenuate TGF-*β*1-induced fibrosis in rat fibroblasts and attenuate induced pulmonary fibrosis [16]. The active ingredient of safflower (hydroxysafflor yellow A) can decrease the expression level of the TGF-*β*1 protein in lung tissue and inhibit bleomycin-induced pulmonary fibrosis [17]. Treatment with safflower yellow significantly downregulated cell proliferation, migration, and the expression of p-ERK1/2, AP-1, collagen I, and collagen III. Safflower yellow exhibits anti-proliferative, anti-migratory, and pro-apoptotic activities in rat aortic adventitial fibroblasts [18].

## 5. Conclusions

In this experiment, a rat peritoneal fibrosis model was used to simulate patients with peritoneal fibrosis caused by long-term peritoneal dialysis. This experiment has proven that the Niao Du Kang mixture can effectively protect the residual renal function of model rats and reduce the peritoneal thickness and the degree of fibrosis. The Western blot and RT-PCR results suggested that the Niao Du Kang mixture might protect the morphological structure of peritoneal mesothelial cells, inhibit the EMT process of peritoneal epithelial cells, and protect the peritoneal function of peritoneal dialysis patients through influencing the Wnt/*β*-catenin signaling pathway on EMT. In conclusion, the Niao Du Kang mixture may inhibit the EMT process of peritoneal epithelial cells through the Wnt/*β*-catenin signaling pathway.

## Figures and Tables

**Table 1:** The effect of Niao Du Kang mixture on the levels of Scr and 24 h-Upro in rats with peritoneal fibrosis ($\bar{x}\pm s$, *n* = 10).

Group	Dosage	Scr (*μ*mol/L)	24 h-Upro
Sham operation	—	30.75 ± 1.98	0.34 ± 0.16
Model	—	135.57 ± 62.90^∗∗^	5.63 ± 4.16^∗∗^
Niao Du Qing granules	2.5 g/kg	63.89 ± 9.37^##^	1.05 ± 0.76^##^
Niao Du Kang mixture	20 ml/kg	59.50 ± 8.49^##^	1.03 ± 1.15^##^
	10 ml/kg	70.56 ± 13.40^##^	2.20 ± 1.86^##^
	5 ml/kg	73.86 ± 19.15^##^	1.83 ± 1.93^##^

*Note.* Compared with the sham operation group,^∗∗^*P*  <  0.01; compared with the model group, ^##^*P*  <  0.01.

**Table 2:** The effect of the Niao Du Kang mixture on the peritoneal thickness of peritoneal fibrosis rats ($\bar{x}\pm s$, *n* = 10).

Group	Dosage	Peritoneal thickness (*μ*m)
Sham operation	—	13.16 ± 1.61
Model	—	53.09 ± 6.15^∗∗^
Niao Du Qing granules	2.5 g/kg	28.98 ± 8.20^##^
Niao Du Kang mixture	20 ml/kg	28.97 ± 7.10^##^
	10 ml/kg	21.15 ± 7.19^##^
	5 ml/kg	27.52 ± 8.43^##^

*Note.* Compared with the sham operation group, ^∗∗^*P* < 0.01; compared with the model group, ^##^*P* < 0.01.

## Data Availability

The data used to support the findings of this study are included within the article.

## Supplementary Materials

Supplementary Materials The corresponding cell experiments have been completed, and the experimental results showed that Niao Du Kang mixture can reduce the degree of EMT in peritoneal mesothelial cells HMRSV5 cells. Supplementary cell experiment to this article can be found in supplemental files.
