# Supplementary material for: Intervention Mechanism of Niao Du Kang Mixture on the EMT Process of Peritoneal Fibrosis Based on the Wnt/β-Catenin Signaling Pathway
Source: Evid Based Complement Alternat Med. 2022 Nov 7;2022:2089483. doi: 10.1155/2022/2089483 (PMC9663227; doi:10.1155/2022/2089483)
Supplement: Supplementary Materials — The corresponding cell experiments have been completed, and the experimental results showed that Niao Du Kang mixture can reduce the degree of EMT in peritoneal mesothelial cells HMRSV5 cells. Supplementary cell experiment to this article can be found in supplemental files. [file 2089483.f1.docx]

# Cell Experiment

## 1 Materials

### 1.1 Drugs and cells

Tested drug: Niao Du Kang mixture (as per animal experiment); cell line: human peritoneal mesothelial cell (HPMCs) HMrSV5 ( [Shanghai Hongshun Biotechnology Co., Ltd.](https://www.bio-equip.com/supplyinfo65431.html) )

## 2 Methods

### 2.1 HMRSV5 cell recovery, passage, and cryopreservation

Take out the cryopreserved HMRSV5 cells from liquid nitrogen, lyse, centrifuge, resuspend the cells with DMEM/F12 containing 10% FBS, 100U/ml penicillin, and 100U/ml streptomycin, add medium, and culture in a constant temperature incubator. The cells were passaged when the growth density reached 80%. Cells were digested with 0.05% trypsin (0.25% trypsin diluted in PBS) for 5 minutes. The digestion was terminated with DMEM/F12 medium containing 10% FBS, then the cell suspension was collected, centrifuged, and the cells were resuspended in the medium, passaged at a ratio of 1:3, and cultured in a constant temperature incubator. The cell suspension was placed in a cryopreservation tube, and the cells were cryopreserved by programmed cooling.

### 2.2 Preparation of medicated serum of Niao Du Kang mixture

6 times of the clinical equivalent dose of Niao Du Kang mixture, i.e. 8.57ml/kg, administered once in the morning and evening, 8.57ml/kg each time. After administration for 5 days, the serum was extracted, inactivated at 56℃ for 30 minutes, filtered with a 0.2 μm filter membrane and stored at -80 °C for later use.

### 2.3 LPS-induced fibrosis of HMRSV5 cells in renal tubular epithelial cells

HMRSV5 cells were cultured by Glucose ( G ) (25 μM) and LPS (10 μg/ml) for 72 h ,to induce fibrosis in HMRSV5 cells, and the cell pellets were collected and stored at -80 °C for later use.

### 2.4 Real-time PCR, Western blot detection in changes of LEF-1, E-Cadherin, β-catenin, collagen 1, Wnt-1, α-SMA mRNA and protein expression

Use protein loading buffer 5X to treat protein samples, and quantified according to the protein concentration. The loading volume of each well was 10 μl containing 30 μg of protein samples, filled with PBS, and the protein was denatured in a water bath at 100 °C for 10 min. Trim the 0.45 µm PVDF membrane and soak in transfer buffer for 15 minutes; assemble the sandwich in the order of filter paper, gel, PVDF membrane, and filter paper; assemble into a trarsmembrane sandwich , place it in the transfer tank with constant current for 2 hours, then washed, enclosed for 1 hour at

Shaker and room temperature. Proportionally dilute antibodies of LEF-1, E-Cadherin, β-catenin, collagen 1, wnt-1, α-SMA. Incubate overnight. The secondary antibody (HRP-labeled goat anti-mouse or goat anti-rabbit IgG antibody) is determined according to the source of the primary antibody, diluted with 1×TBST at 1:2000, shaker at room temperature, incubated, shaker with 1×TBST, washed for 5 minutes for 3 time; Image development and band analysis: ECL imaging system with luminescence development, Image J software analyzes the gray value of the band, and uses t-test or analysis of variance for statistical analysis.

## 3 Results

mRNA expressions of E-Cadherin, α-SMA, Collagen - Ⅰ, β-catenin , Wnt -1 and LEF-1 in the G+LPS group were higher than those in the blank group, and the Niao Du Kang medicated serum group was significantly lower than that in the G+LPS group ( *P* < 0.05 , *P* < 0.01 ). See Figure a.

The protein expressions of E-Cadherin, α-SMA, Collagen -Ⅰ, β-catenin , Wnt -1 and LEF-1 in the G+LPS group were significantly higher than those in the blank group ( *P* < 0.05 , *P* < 0.01 ). The protein expressions in the Niao Du Kang medicated serum group was significantly decreased compared to the G+LPS group (*P* < 0.05, *P* < 0.01). The protein expressions of E-Cadherin , α-SMA, Collagen - Ⅰ , β-catenin, Wnt-1 and LEF-1 in Gallocyanine group decreased ( *P* < 0.05 ), while those in Gallocyanine + Niaodukang group have increased compared to the blank group ( *P* < 0.05 ) . See Figures b and c.

Note: compared to the blank group, *^*^ P* <0.05 , *^**^ P* <0.01; compared to the G+LPS , *^##^ P* <0.05 , *^##^ P* <0.01.

Figure a , PCR statistical results


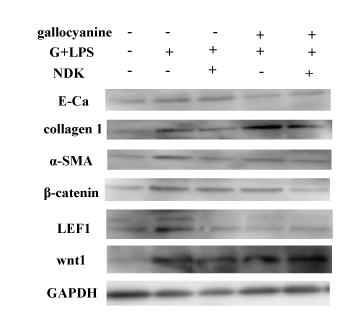


Figure b

Note: compared to the blank group, *^*^ P* <0.05 , *^**^ P* <0.01; compared to the G+LPS , *^##^ P* <0.05 , *^##^ P* <0.01.

Figure c, gray value statistical results

**conclusion**

Niao Du Kang mixture can reduce the degree of EMT in peritoneal mesothelial cells HMRSV5 cells .
